# The Toxicological Mechanisms of Environmental Soot (Black Carbon) and Carbon Black: Focus on Oxidative Stress and Inflammatory Pathways

**DOI:** 10.3389/fimmu.2017.00763

**Published:** 2017-06-30

**Authors:** Rituraj Niranjan, Ashwani Kumar Thakur

**Affiliations:** ^1^Department of Biological Sciences and Bioengineering (BSBE), Indian Institute of Technology Kanpur, Kanpur, India

**Keywords:** soot (black carbon), carbon black, inflammation, oxidative stress, polyunsaturated fatty acids, air pollution

## Abstract

The environmental soot and carbon blacks (CBs) cause many diseases in humans, but their underlying mechanisms of toxicity are still poorly understood. Both are formed after the incomplete combustion of hydrocarbons but differ in their constituents and percent carbon contents. For the first time, “Sir Percival Pott” described soot as a carcinogen, which was subsequently confirmed by many others. The existing data suggest three main types of diseases due to soot and CB exposures: cancer, respiratory diseases, and cardiovascular dysfunctions. Experimental models revealed the involvement of oxidative stress, DNA methylation, formation of DNA adducts, and Aryl hydrocarbon receptor activation as the key mechanisms of soot- and CB-induced cancers. Metals including Si, Fe, Mn, Ti, and Co in soot also contribute in the reactive oxygen species (ROS)-mediated DNA damage. Mechanistically, ROS-induced DNA damage is further enhanced by eosinophils and neutrophils *via* halide (Cl^−^ and Br^−^) dependent DNA adducts formation. The activation of pulmonary dendritic cells, T helper type 2 cells, and mast cells is crucial mediators in the pathology of soot- or CB-induced respiratory disease. Polyunsaturated fatty acids (PUFAs) were also found to modulate T cells functions in respiratory diseases. Particularly, telomerase reverse transcriptase was found to play the critical role in soot- and CB-induced cardiovascular dysfunctions. In this review, we propose integrated mechanisms of soot- and CB-induced toxicity emphasizing the role of inflammatory mediators and oxidative stress. We also suggest use of antioxidants and PUFAs as protective strategies against soot- and CB-induced disorders.

## Introduction

The environmental soots [black carbon (BC)] and carbon blacks (CBs) cause many health issues in humans and animals (1, 2). The terms soot and CB have been used interchangeably but, both are physically and chemically distinct entities (3–5). Soots are considered as unwanted byproducts derived from incomplete combustion of carbon-containing materials (3–5). In contrast, the CBs are manufactured under the controlled conditions in the rubber, printing and painting industries for commercial use (3–5). Soot is a powdery mass of fine black particles (6–8). It consists of impure carbon, formed after the incomplete combustion of hydrocarbons (9). The main source of environmental soot is the combustion of fossil-based fuels and biomass burning at the Earth’s surface (10). The other examples of soot may include coal, charred wood, petroleum coke, cenospheres, and tars (11, 12). To a smaller extent, quartz/halogen bulbs with settled dust, cooking, oil lamps, smoking of plant matter, fireplaces, candles, house fires, furnaces, and local field burning also contribute to the soot production (13). Soot particles range from about 10 nm to 1 mm in size (14–17). The relative amount of elemental carbon inside soot is considered to be less than 60% of the total mass of particle (4, 7, 18). Among hydrocarbons, the poly aromatic hydrocarbons (PAHs) are the main carcinogenic compound in the soot (19–21). At elemental level, the most characterized diesel soot contains carbon (as a main component), hydrogen, oxygen, sulfur, and trace amount of metals (22–24). The major component of soot, the BC, causes premature human mortality and disability (25). Furthermore, changes in the chemical composition of soot are accomplished due to heterogeneous oxidation reactions in the environment (26–28). From the regional point of view, developed nations were the biggest source of soot (BC) emissions but at the present scenario, soot emissions are majorly from developing countries (29, 30). It is noted that United States emits about 6.1% of the world’s soot (31). Notably, the biggest amount of soot comes from Latin America, Asia, and Africa (31, 32). India and China alone may account around 25–35% of total global soot emissions (31–34). The impact of soot on the human health and to the entire environment depends on its distribution and its distance from the source of origin (35–38). Soot from vehicle exhausts comes from combustion of diesel, gasoline, and other petroleum-based fuels materials that contains carbonaceous particles, having polycyclic aromatic hydrocarbons (PAHs) attached to it (21). Typically, diesel exhaust particles (DEP) are made up of carbon core with some volatile and semi-volatile (such as H_2_SO_4_ and organics) components adsorbed on it (9, 39). It has been believed that the vehicle exhaust contributes to approximately 50% of urban particulate matter (PM) (40). The special attention is given to the smaller fractions of PM (PM 2.5 and PM 0.1) because these particles can penetrate deep into the bronchiolar parts of the lungs and cause various health hazards (41).

**Figure 1:**
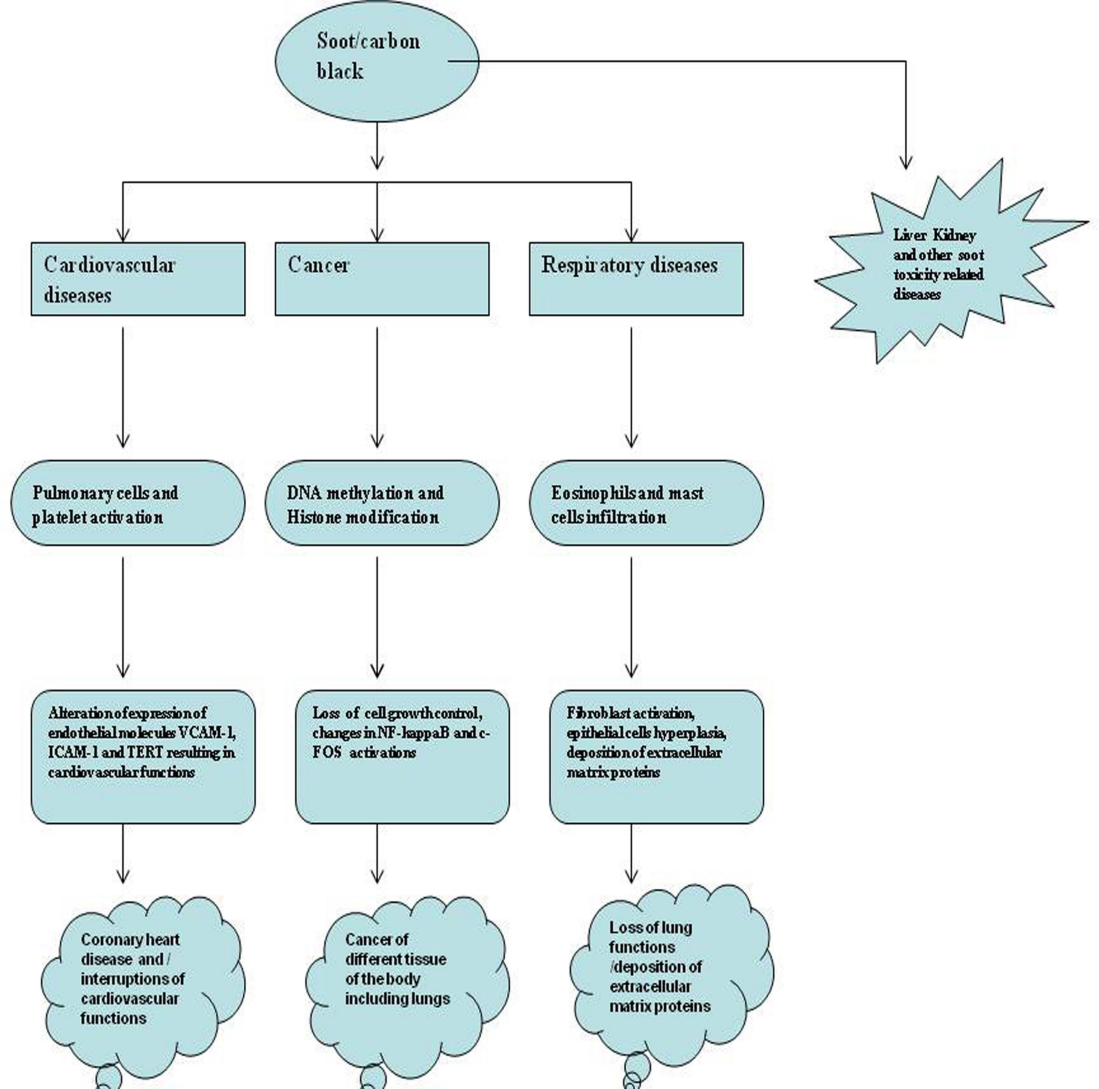
The major health problems due to soot and carbon black (CB). Figure shows soot- and CB-induced major health problems. The first hazard is cancer that is caused by DNA adducts formation, DNA strands breaks, or mutation in genes. Second is the respiratory toxicity caused by dysfunctional immune response involving activation of eosinophils and mast cells. The third is cardiovascular toxicology that also includes the coronary heart disease. Apart from these, soot also causes damage to the different organs of the body by some unknown mechanisms.

In contrast to soot (BC), CB is generated by the partial combustion of heavy petroleum materials such as coal tar, ethylene cracking tar, and FCC tar (42). The common subtypes of CB are furnace black, acetylene black, lamp black, channel black, and thermal black (3, 43). CB is also known by the trade names such as Printex-90, Printex-140, Printex-G, and Lampblack-101 (3, 43). Around 95% of CB production is achieved by the oil furnace process (3, 44, 45). In this process, the heavy aromatic petroleum products are pyrolyzed at the very high temperatures (around 1,400–1,800°C) to make the CB particles and tail gas (e.g., carbon monoxide, hydrogen, and steam) (46, 47). This process is conducted as a continuous process in a closed reactor. It is interested to know that the production is highly controlled in the oil furnace process, such that various CBs with the differing properties can be made (3, 46). The amount of elemental carbon is greater than 97% in CB that is arranged as aciniform-like structures. CB has widely been used to produce both *in vitro* and animal models of soot toxicology and will be discussed in detail in latter sections of this article (3–5). CB is also dissimilar to environmental soot especially due to its higher surface area to volume ratio as well as very less (less bioavailable) polycyclic aromatic hydrocarbon contents (3). Importantly, both soot and CB mainly affect cardiovascular system, respiratory system, and cause different kinds of cancer (Figure 1) (41). Therefore, it is important to know soot- and CB-induced toxicity in these major disease areas.

## Historical Perspective of Soot- and CB-Induced Health Effects

**Figure 2:**
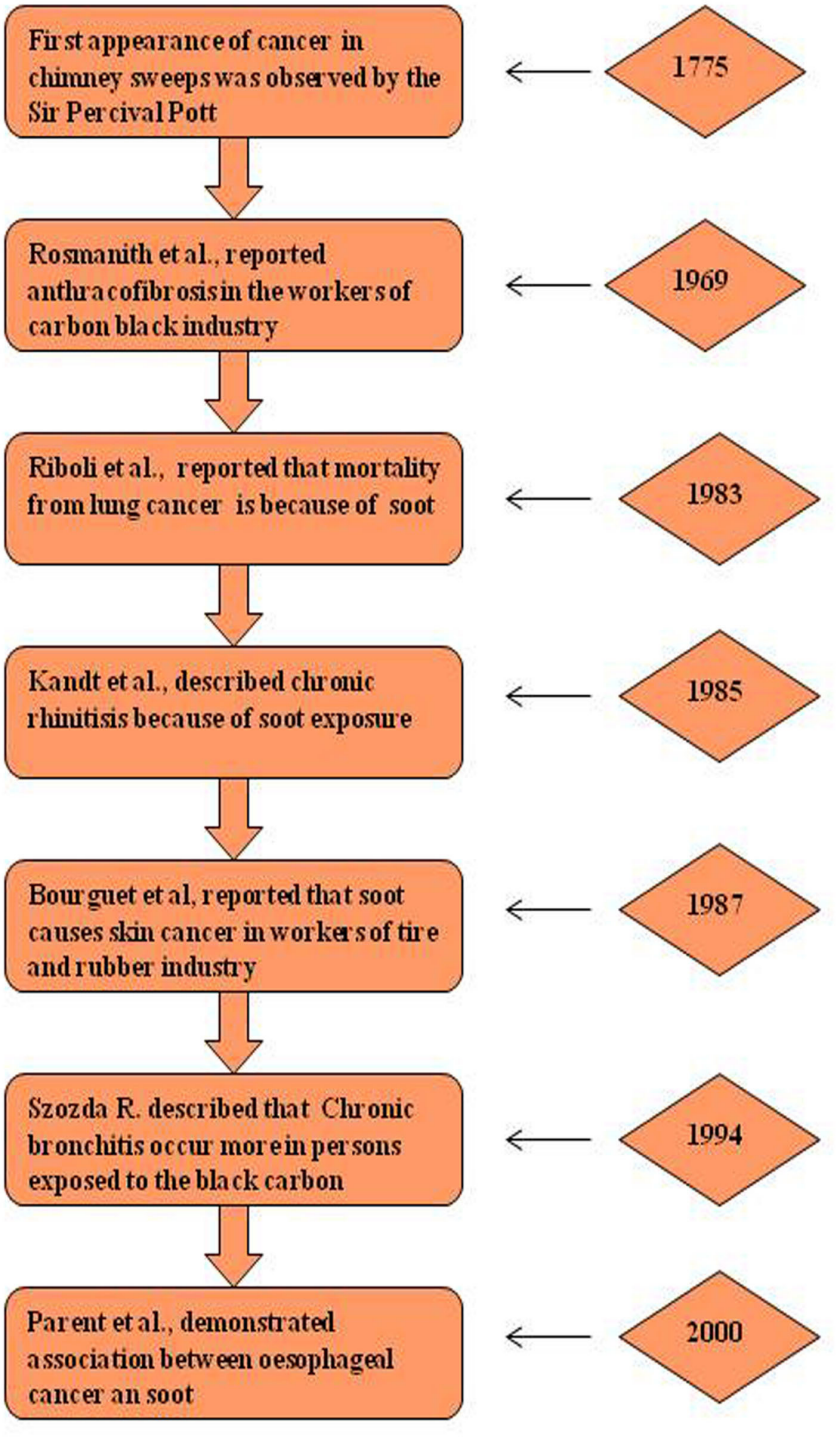
Historical perspective of soot-induced health hazards. Diagrammatic representation of major breakthrough studies due to soot and carbon black exposure (62). The left panel shows the pathological manifestation and right panel shows the corresponding year of study.

**Figure 3:**
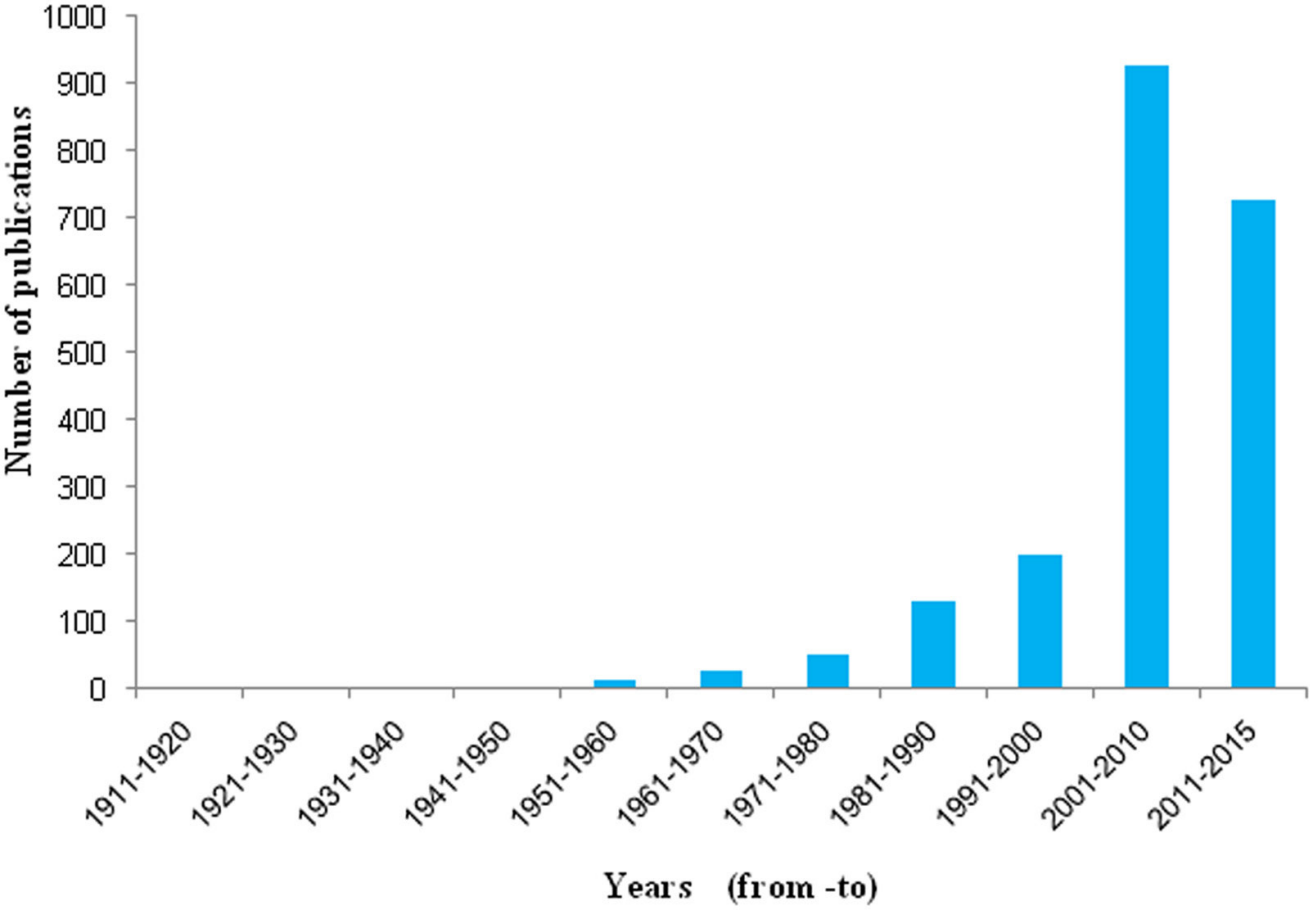
Number of publication found in PubMed related to soot. Histograms represent decade wise publications in the area of soot toxicity, collected from PubMed search using the word *soot*.

Historically for the first time, Sir Percival Pott, a London surgeon, in 1775 recognized that chimney sweeps were particularly susceptible to develop scrotal cancer. He attributed this disease to the soot exposure to workers (Figure 2) (48, 49). Later he described soot, as the first environmental factor to cause cancer. This linkage started the chain of events that led to the development of first experimental model of cancer and the synthesis of first carcinogen (49, 50). Later, Earle and Paget confirmed soot, as a general human skin carcinogen (48, 51). In 1936, the proof indicating soot as a carcinogen was first given by the findings of Kuroda and Kawahata (50, 51). In the year of 1969, Rosmanith et al. reported anthracofibrosis (characterized by the luminal narrowing and black pigmentation in the mucosa) in the workers of CB industry (52). Further in 1983, Riboli et al. have reported the mortality from lung cancer in the individuals working in the manufacturing plant of acetylene and phthalic anhydride, due to soot exposure (53). Subsequently, Snow found that the inhalation of CB leads to its accumulation into the larynx and trachea resulting into multiple disease situations (54). Kandt and Biendara (55) observed the appearance of chronic rhinitis more frequently in soot-exposed workers than in unexposed persons (55). Beck et al. (56) further confirmed that the soot exposure leads to causation of cancer (56). In 1987, Bourguet et al. considered soot as a major factor for cancer of skin in the persons working in the tire and rubber industry (57). Another study by Parent et al. was conducted in 1996 to find a relationship between exposure of CB and lung cancer risk assessment in a population-based study in Montreal, QC, Canada. This study provided additional support for the fact that exposure to CB leads toward the development of lung cancer (58). In 1994, Szozda described frequent occurrence of chronic bronchitis and ventilation disturbances in persons exposed to the BC (59). Recently, Parent et al. (60) demonstrated an association between esophageal cancer and in occupational exposures of sulfuric acid and CB (60). These evidences in the history clearly show the association of soot and its constituents to human health, but its exact mechanism of toxicity remains elusive and need further experimentation, both at epidemiological and animals levels (61). Nevertheless in the last decade, there is a significant increase in the number of soot and CB toxicity studies that will be discussed in detail (Figure 3).

## Major Diseases and Rare Pathological Manifestations Due to Soot and CB

Over three centuries, the linkages of soot and CB with different diseases have been observed (25). Soot and CB cause many diseases but only three are understood to some details (Figure 1). The more complex disease associated with the soot and CB is the occurrence of cancer. Soot- and CB-induced cancers are localized and systemic in nature (34). The second major health issue with soot and CB is respiratory disorders, which sometimes can be very severe. The third one is the cardiovascular dysfunctions. Apart from these diseases, some unique pathological observations have also been seen in response to soot or CB exposures (Table 1). In a study, prenatal exposure of pritex-90 caused sexual and neuroinflammatory changes in mice (63). Surprisingly, lung exposure of diesel engine exhaust significantly influenced pro-inflammatory markers of the rat brain (64). In another study, Printex-90 lowered the sperm production (65). Similarly, carbon nanoparticles were found to adversely affect the male reproductive system of mice (66). Recently, it was known that CB exerts developmental toxicity by the immune activation in the male offspring of mice (67). Soot from a transformer fire was also seen to induce salivary gland duct metaplasia in guinea pigs (68). These studies show the involvement of systemic response of the body in the development of different pathologies that further need extensive exploration.

**Table 1:** Soot- and carbon black (CB)-induced special pathological manifestations in different experimental models.

Form of soot/CB	Concentration/dose used	Animal models	Parameters studied	Results and interpretation	Reference
Soot from a transformer fire	46.3 ppm for 90 days	Guinea pig	Salivary gland morphology	Salivary gland duct metaplasia	(68)
Diesel engine soot	Nasal exposure	Rat	Inflammatory mediators in the brain	Soot-induced inflammation is liked with brain pathology	(64)
					(69)
CB (Printex-90)	67 μg/animal	Female mice	Expanded simple tandem repeat (ESTR) germline mutation rates	ESTR mutation rates were not statistically different	(63)
CB (Printex-90)	268 µg/animal	Female mice	Sexual development and neurofunction	Sexual development and neurofunction were altered	(65)
CB	0.1 mg/mouse for 10 times every week	Mice	Male reproductive system	Serum testosterone levels were elevated	(66)
CB (Printex-90)	NA	Mice	Sperm production	Showed lowered sperm production	(70)
CB or diesel exhaust particles	300 µg/m^−3^	Mice	Heart rate (HR)	HR is significantly decreased	

Although soot and CB cause similar effects, but there is distinctions in their effects from the structure and composition point of view (71). It has been observed that the CB exposure in some studies has lees carcinogenic effect than the environmental soot exposure (Table 2) (21). It is believed that poly aromatic hydrocarbons exist in the CB are not biologically accessible as compared to the soot (BC) (46, 71, 72). As CB is formed under the controlled conditions, the bound poly aromatic hydrocarbons are less bioavailable compared to soot (BC). In the next sections of this article, three major diseases will be discussed in detail. At the beginning of each section, the epidemiologic studies and clinical manifestations will be discussed. Subsequently, findings from the animal models will be described.

**Table 2:** Examples of studies with carbon black (CB) where it fails to cause pathology.

Key findings of study	Associated disease	CB used	Reference
Occupational exposure to CB did not experience any detectable excess risk of lung cancer	Cancer	CB	(73)
Aryl hydrocarbon receptor activation was observed by particulate matter, which was responsible for the antiapoptotic effects, however, CB fails to do so	Cancer	CB	(74)
*In utero* exposure of nano-sized CB did not induce tandem repeat mutations in germ cells	Cancer	CB	(69)
The CB exposure did not induce acute phase response in the liver (the production of serum amyloid A proteins, Sap, Saa1, Saa3)	Acute phage response serum amyloid	CB	(66)
Intratracheally administration of CB did not aggravate elastase-induced pulmonary emphysema in rats	Pulmonary emphysema	CB	(75)
CB did not induce impairment of NO-dependent relaxation in intralobar pulmonary arteries	Cardiovascular disease (relaxation of arteries)	CB	(76)

## The Pathological Mechanisms of Cancer Due to Soot and CBs

As introduced in the previous section, soot is the first known carcinogen responsible for the development of different types of cancer in humans and experimental models. These cancers may have local or distal appearance from the site of exposure (48, 50, 77). It was noted that despite the efforts of 200 years to control the safety in the soot-related work, chimney sweeps still show increased mortality from cancer (78). In line of this, a case report from Gerber described that the development of penis carcinoma in chimney sweeps was caused due to soot exposure (79). In Swedish chimney sweeps, the cancer excess was also reported due to soot and asbestos exposure (80). Soot is absorbed and transported to blood by airway epithelium and majority of the cancers in the distal body parts may be accompanied due to this mechanism of soot transportation (81). A population-based study showed that, occupational exposures to polycyclic aromatic hydrocarbons, a component of soot is responsible for respiratory and urinary tract cancers (82). Another case–control study in rubber manufacturing industry showed CB as a major contributor to the early cancer of skin (57). Subsequently, it became clear that exposure to polycyclic aromatic hydrocarbons (PAHs) in diesel soot are responsible in the development of prostate cancer (83). Furthermore, CB nanoparticle exposure-mediated human health risk was confirmed by gene expression profiling (84). Contradictory to the other studies, the International Agency for Research on Cancer (IARC) in Montreal, QC, Canada reported that the subjects with occupational exposure to titanium dioxide, industrial talc, CB, and cosmetic talc did not experience any detectable excess risk of lung cancer (85). However, this study was limited to the lung pathology alone, and no other organs were investigated (85).

**Figure 4:**
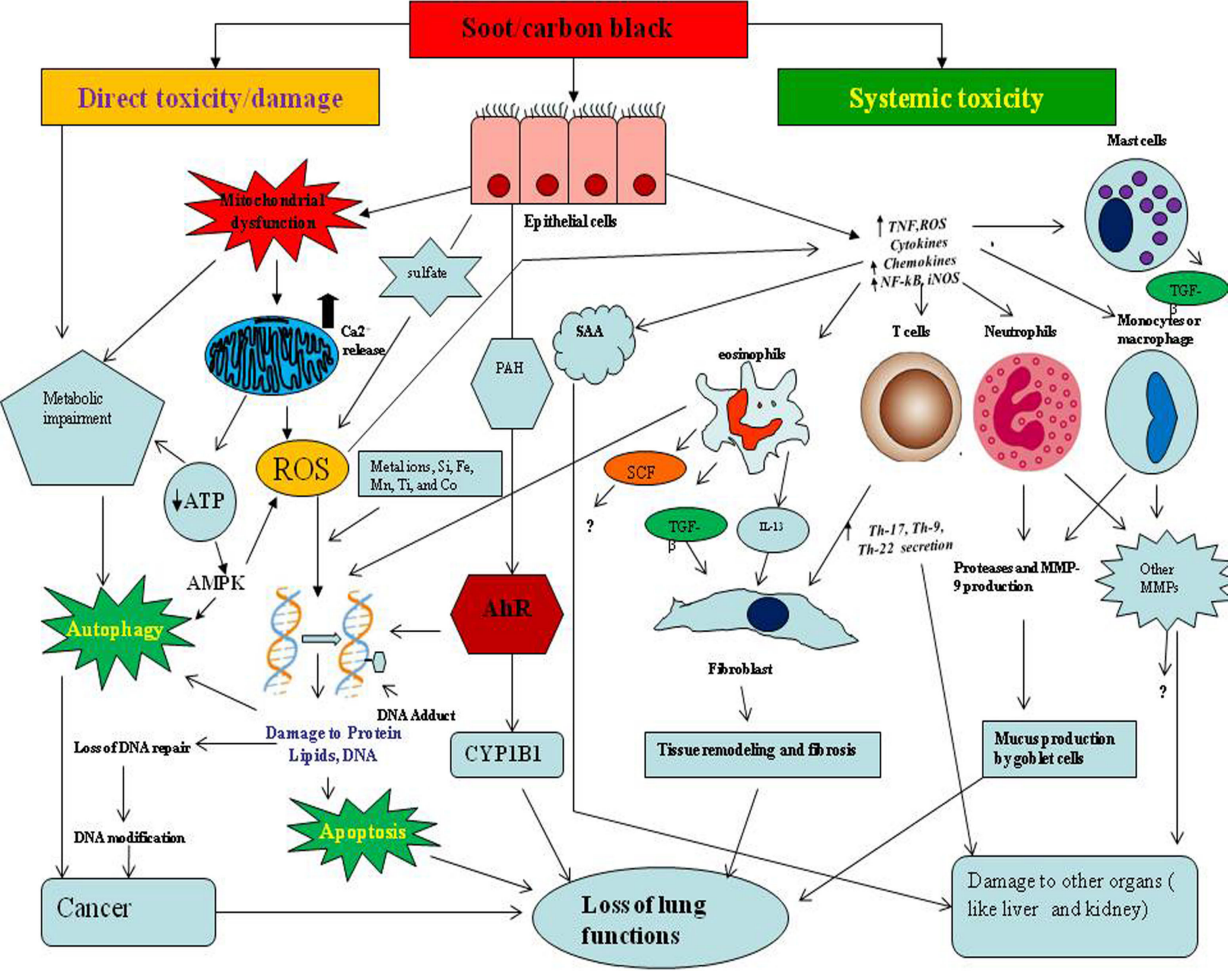
The proposed mechanism of soot- and carbon black (CB)-induced toxicity. The toxicological mechanisms of soot or CB can be theoretically classified into two types. The one is direct toxicity or localized damage, and the other is systemic toxicity. In direct toxicity, soot/CB comes into contact with lung epithelial cells, produces oxidative stress by affecting mitochondria, and upregulates calcium influx in the cells. The soot-induced oxidative stress initiates the cell survival or death mechanisms such as autophagy and apoptosis. Soot may lead to cancer development by interruption of autophagic and apoptotic cell death. Soot or CB also induces DNA methylation, DNA adducts formation, Aryl hydrocarbon receptor (AhR) activation, DNA double-strand breaks, and failure to DNA repair mechanisms, resulting in cancer. In systemic toxicity, soot triggers an inflammatory response in lungs and causes various symptoms. Due to soot or CB exposure, lung epithelial cells secrete inflammatory mediators (chemokine and cytokines), which further amplify the immune response. Immune cells produce interleukin-13 (IL-13) and transforming growth factor beta (TGF-beta) which activate fibroblast cells to acquire fibrotic phenotype (production and accumulations of fibrous proteins in extracellular space). Monocytes and macrophage produce a large number of matrix metalloproteases (MMPs) and other toxic mediators that alter many physiologic functions including brain and cardiovascular systems. Serum amyloid A (SAA) produced in response to soot causes problems to the liver and kidney.

In addition to the above described evidences, experimental models also provided data to further support the cancer causing properties of soot and CB (Figure 4). Study conducted on dogs demonstrated that the absorption of soot through alveolar epithelium is means of entry to the circulation of un-metabolized PAHs (86). It has been shown in rat model that soot particle interactions with lung tissue is responsible for morphological changes in the lungs (87). The diesel exhaust (DP) and CB when regularly inhaled by rats showed toxic and pulmonary carcinogenic properties (88, 89). *In vitro* study on the carcinogenic potency of CB confirmed the genotoxic basis of soot toxicity (90).

At molecular level, DNA methylation changes occur after the exposure of ambient particulate pollutants (PM, BC) with aerodynamic diameter ≤2.5 µm [PM_2.5_], which in turn alter the expression profile of genes toward the development of cancer (Figure 4) (91). Interestingly, polycyclic aromatic hydrocarbons can alter the histone modification and therefore are responsible for epigenetic effects (92, 93). Another study reported that soot and CB cause genotoxic effects by making single- and double-stranded DNA breaks (92, 93). DEP enhanced nuclear factor kappa B (NF-kB) DNA-binding activity along with alteration profile of c-FOS proto-oncogene expression in cultured human epithelial cells, indicating the initiation of cancer (94). Notably, butadiene soot (a PAH-containing soot) exposure increases the of matrix metalloproteases-9 (MMP-9) protein expression in prostate cancer cell line. This indicates that hydrocarbons can also stimulate MMP-9 protein secretion that contributes in the development of cancer *via* inflammatory pathway (95). It was found that gene expression modulation occurs in response to combustion-derived nano-sized particles in A549 cells (96). This study demonstrated that several radiation-responsive genes such as GADD45beta and CDKN1A as well as NF-kappa B-dependent genes are altered due to the exposure of nano-sized combustion-derived particles (96).

The ability of soot particles to cause mutations has long been debated, but recent reports show that soot particles indeed can cause DNA mutations (97). Soot particles from the 1991 oil fires of Kuwait desert were studied for its property to induce genetic effects in *in vitro* conditions (98). In this study, dose-dependent increase was seen for both sister chromatids exchanges in peripheral blood lymphocytes. Also, a mutation at the hprt locus was observed in the metabolically competent AHH-1 cell line (human lymphoblast cell line) in response to soot (98). It was further confirmed in an *in vitro* study of cultured cells that the soot causes the mutation in the DNA and induces genotoxic effect (97). CBs also induced genotoxic effects by damaging DNA in presence of magnetite (Fe_3_O_4_) and polycyclic aromatic hydrocarbons (PAHs) (99). However, *in utero* exposure of nano-sized CB (Printex-90) did not cause mutations (tandem repeat) in germ cells (69). The one of the crucial mechanisms that causes DNA modification is the DNA adducts formation due to soot or CB exposure (100, 101). The chemical modification of DNA occurs when, polycyclic aromatic hydrocarbons (PAHs) reacts to the DNA molecule and form DNA adducts. In line of this, an important study described that chronic inhalation of diesel emissions and CB caused DNA adducts formation in the rat lungs (102). The formation of 8-oxo-7,8-dihydro-2′-deoxyguanosine in the DNA of rat lungs following sub-chronic exposure of CB (Printex-90) was also found (103–105). Subsequently, it was elucidated that soot and/or CB exposure induced oxidative stress in associated with the formation of DNA adducts *via* a failure of DNA repair mechanism (106). Mechanistically, it was observed that reactive oxygen species (ROS) enhance DNA damage by inflammatory cells such as eosinophils and neutrophils *via* the helide (Cl^−^ and Br^−^)-dependent formation of DNA adducts and thus enhancement of cancer (107). It is already evident that sulfate (a semi-volatile component) present in the soot particles directly induces oxidative stress in the cells (108, 109). It is also found that sulfate in DEP may involve in the tissue remodeling and fibrosis (110). Another important mechanism that may play a key role at molecular level is the aryl hydrocarbon receptor (AhR) activation and DNA damage (101, 104, 111, 112). It was evident that soot nanoparticles significantly upregulate the AhR activation in the lungs, which may subsequently influence the oxidative damage and inflammation (113, 114). Soot also binds with the AhR receptor and may affect at cells and organ levels but the mechanism of its interaction to AhR receptor is not yet known (115). It was found that the activation of AhR *via* air pollutants can induce inflammation and subsequent allergic diseases (116). Activation of AhR induces the secretion of PDGF-BB by activated macrophages when exposed by DEP and stimulates lung fibroblast proliferation (117). Recently, it was confirmed that AhR activation and Nrf2 activation are the key mechanism in the induction of oxidative stress in response to air PM (soot) exposure (118). The AhR-mediated functions were also observed due to PM exposure, such as AhR-dependent cell proliferation and cytochrome P450 1A1/1B1 expression (119). The metals in PM2.5 were associated in the activation of AhR that subsequently influence the pathogenesis of infections in child (120). It was also observed that poly aromatic hydrocarbons found in PM are responsible for the AhR-mediated antiapoptotic effects where CB fails to do so (72, 74, 121).

It is also believed that trace amount of metal ions present in the soot are responsible for its toxic effects (122–124). A significant correlation in the metal content (Si, Fe, Mn, Ti, and Co) present in the ambient air samples was seen with the unregulated ROS formation in polymorphonuclear leukocytes (124–126). It was further proved that metal ions enhance the ROS formation capacity of ultrafine black particles both *in vivo* and *in vitro* (127). In has now been clear that metal ions present in the DEP are responsible for its genotoxic effects (128). A recent study described the estrogenic activity of soot is not related to the metal ion concentrations present in it (129). In soot, mainly BC and metal ions are responsible for the ROS generation in lung cells (130). Another study described that intracellular calcium ion formation and inflammation induced by ultrafine carbon black (ufCB) is not dependent on metal ions and other components. Collectively, sufficient amount of data support the claim that soot and CB are able to cause cancer *via* the genetic and molecular modifications.

## The Mechanisms of Pathophysiology in Respiratory Diseases Due to Soot and CB

The respiratory epithelium of the lungs is the first tissue to get constant exposure with different kinds of soots and CBs present in the environment. Soot or CB toxicity causes the interruption of respiratory process by alteration in lung functions (131). These toxicological mechanisms may be of two kinds. The first mechanism is the direct contact-mediated dysfunctions of lung cells that include ROS generation, cell hyperplasia, cell death, or apoptosis of lung airway epithelium and other adjacent cells (Figure 4) (132). The second mechanism includes the involvement of systemic immune response resulting in the development of tissue remodeling and fibrosis that causes problem in breathing and lung dysfunctions. In this section, we would discuss these two types of toxicities caused by soot or CB in the context of human clinical and animal studies.

The two respiratory diseases that are mainly reported in humans due to soot exposure are chronic obstructive pulmonary disease (COPD) and asthma (133). The pathophysiology of asthma involves the inflammation of airways, tissue remodeling and fibrosis, obstruction of airflow intermittently, and hyper responsiveness of bronchi (134, 135). The pathophysiology of COPD includes airway inflammation, mucociliary dysfunctions, and structural changes (136, 137). There are numerous evidences that support the linkage of soot or CB with asthma and COPD. A study reported that an early exposure to the air pollution leads to the development of childhood asthma (138). Ultrafine particles (UFPs) (soot) and carbon monoxide concentrations are associated with asthma enhancement in the urban children (139). DEP initiate the alveolar epithelial cell movement by alterations of polarity mechanisms (140). An epidemiological study reported that healthy subjects were affected by agriculture crop burning with their altered peak expiratory flow rate and pulmonary functions (1). The patients prone to COPD or asthma already exhibit preexisting oxidative stress and hence are more susceptible toward soot-mediated oxidative damage. Interestingly, it is known that ufCB causes adverse effects *via* ROS and may have worse manifestations in these susceptible persons (141).

The evidences from animal models also supported soot- and CB-mediated mechanisms of toxicity. A rat model of study described that the flame-generated ultrafine soot increased the ROS and upregulated Nrf2 antioxidants in the lungs (142). Similar studies found that the neonatal lungs are more susceptible to ultrafine soot as compared to adults (143). The ultrafine soot also generates ROS and induces DNA damage (144). Moreover, CB enhanced ROS in the rat alveolar macrophages, this is an example where non-biodegradable components of CB can generate an immune and oxidative stress response (145). Another study showed excessive generation of ROS by monocytes upon exposure of CB (146). It is already known that increased production of pro-inflammatory mediators are linked with the activation of specific transcription factors such as NF-kB, through the Ca^2+^ upregulation and ROS formation (146–148). A study suggested that ufCB triggers an increase in cytosolic Ca^2+^, possibly through entry of extracellular Ca^2+^ *via* the Ca^2+^ channels in the plasma membrane (146). Therefore, nanoparticles activate the opening of Ca^2+^ channels by means of ROS (146). It has now been unrevealed that alterations in the glutathione and superoxide dismutase activities are the key enzymatic mechanisms involved in the generation of oxidative stress by CB (149). CB induced ROS that involves lot of enzymatic reactions including ERK MAP kinase cascade pathway (145). NADPH quinone oxidoreductase-1 enzyme was also activated following DEP exposure and mediates activation of ROS (150). Interestingly, an antioxidant ceruloplasmin was found upregulated in epithelial cells of lung due to ufCB exposure (151). This emphasizes that antioxidant machinery is triggered in response to ufCB exposure in the lung (151). Increased nitrative stress has also been recently reported to cause DNA damage in response to ufCB particles (152).

Oxidative stress produced by soot or CB is subsequently linked with systemic immune response (inflammation) in the lungs, which results in the development of asthma and other diseases (Figure 4) (153–155). The existing literature supports that the inflammation causes serious damage to the lung functions by many mechanisms, most of which are not properly understood (156, 157). It was found that DEP were taken up by epithelial cells of human airway and altered cytokines production showing inflammation in lungs. These cytokines are known to cause damage to the lung functions (158). Notably, one of the key pathological manifestations of dysfunctional inflammatory response is the development of tissue remodeling and fibrosis. It was seen that soot triggers an inflammatory condition that leads to the accumulation of collagen fibers (56). This study provided the evidence that soot-associated lung inflammation leads to the tissue remodeling and fibrosis (56). In addition to the mediators of inflammation, immune cells also play a very critical role and modulate respiratory functions (33, 159). The Th2-type inflammatory responses and activation of pulmonary dendritic cells were seen on instillation of engineered DEP *in vivo* (160). Similarly, DE enhanced allergen-related eosinophils recruitment to airways and increased protein concentrations of granulocyte macrophage colony-stimulating factor and IL-5 in the lungs of mice (161).

Furthermore, exposure of DEP to rats by intratracheal instillation downregulated LPS-induced TNF-alpha and IL-1 release by alveolar macrophages (162). Similarly, exposure of DEP to rats downregulated the ability of alveolar macrophages to generate the antimicrobial reactive oxidant species in response to zymosan (a fungal component) (163). Moreover, ultrafine carbon particles downregulated cytochrome P450 1B1 expression in human monocytes (164). These data suggested that the Printex-90 decreased the expression profile of CYP gene that may interfere with the detoxification potential of inhaled toxic compounds (164). It is well established that TNF-alpha is a major cytokine responsible for cellular death and causes toxicity (165, 166). The 14-nm CB particles also synergize the ZnCl_2_ stimulated TNF-alpha release (167). Furthermore, zinc-induced morphological changes and cell death were altered by carbon nanoparticles treatment (167). However, there are also some examples where systemic response was not seen. In a study, CB exposure also lacks an acute phase response in the liver (the production of SAA proteins, *Sap, Saa1*, and *Saa3)* (66).

From nutritional point of view, polyunsaturated fatty acids (PUFAs) such as Omega-3 fatty acids or N3-fattyacids have shown protective against asthma risk development (168, 169). Some preclinical studies have shown omega-3 fatty acids as beneficial agents against asthma triggers, such as environmental allergens and viruses (170, 171). The connection with soot toxicity to PUFA is understood on the basis of its antioxidant and anti-inflammatory properties and on its T cell regulatory properties (171). As environmental soot produces plenty of oxidative stress and therefore antioxidant properties of PUFA can act as a protective agents in this area. In patients suffering from respiratory syndrome, a lower level of PUFA and other antioxidants were observed, which are related toward the development of various lung pathologies (172). A high-level PUFA also downregulates oxidative stress-induced chronic bronchopulmonary dysplasia (173). The antioxidant properties of PUFAs also help improve capacity the capacity of exercise in patients suffering from COPD (174). A pilot study described that there have not been any changes in the patients with stable asthma due to *n*-3 PUFAs dietary supplement (175). It is further important to note that there have been few reports that studied the effect of omega-3 fatty acids in the development of asthma pathophysiology (176, 177). Altogether, it can be said that soot and CB affects various biochemical and molecular mediators that in turn cause respiratory dysfunctions.

## The Mechanism of Cardiovascular Dysfunctions Due to Soot and CB Exposures

The cardiovascular diseases due to soot and CB exposure are of major concerns because of their distal appearance from the site of exposure and involvement of more systemic responses (Figure 4). A sufficient amount of clinical and epidemiological data linked soot and CB to cardiovascular dysfunctions. A case-crossover study showed that personal soot exposure is linked with acute myocardial infarction (178). Soot was also seen responsible in the incidence of myocardial infarction (179). In London, air pollution (BC) caused the activation of implantable cardioverter defibrillators (a device used to treat cardiovascular dysfunctions) (180). In Darwin, Australia, the risk of cardiovascular hospitalization was high in people exposed with bushfire particulates (181). Furthermore, air pollution was considered as a major risk factor to the ST-segment (a measure in electrocardiogram) depression in patients suffering from coronary artery disease (CAD) (182). Notably, traffic emission sources of primary organic carbon particles enhanced platelet activation, systemic inflammation, and potentially reduced antioxidant enzyme activity in old people, suffered from CAD (183). It was observed that ufCB particles were associated with accelerated cardiovascular changes, which may compromise “healthy aging” and may trigger cardiovascular diseases (2).

Many studies on experimental models demonstrated the mechanistic basis of soot toxicity leading to cardiovascular dysfunctions (Figure 4). A study revealed that CB affects cardiac autonomous nervous system functions in mice (184). This indicated that the CB can cause the cardiovascular dysfunctions independent of apparent myocardial and pulmonary injury (184). CB nanoparticles exposure also caused endothelial changes *via* modulating nitric oxide synthase expression when it is orally given to the rats (185). The long-term exposure of soot (fine particulate air pollution) was found associated with the adverse cardiovascular outcomes (186). The fact that biodiesel particles are more toxic to health and can cause more cumbersome cardiovascular health issues was shown in a mice model of study (187). In this study, heart rate (HR) and mean corpuscular volume were increased compared with control. Interestingly, leukocytes, reticulocytes, platelets, metamyelocytes, neutrophils, and macrophages were also increased compared with control (187). The involvement of a number of inflammatory mediators along with cells were upregulated in patients with cardiovascular dysfunctions, indicating a role of inflammation in diesel soot-mediated cardiovascular toxicity (187). The myth that UFPs go into the blood circulation was broken by a study showing translocation of UFPs in microcirculation of extrapulmonary organs after the inhalation (188). Moreover, when CB UFPs were infused into intra-arterially in C57BL/6 mice, significant enhancement in platelet on endothelium of post-sinusoidal venules and sinusoids was observed (188). It is already known that immune response is highly regulated by epigenetic mediators and its interactions with endothelial system may cause changes in the cardiovascular system. A study also described that pollution leads to endothelial dysfunctions through epigenetic associations (189). Importantly, ufCB particle changed the expression profile of endothelial nitric oxide synthase in abdominal aorta of animals (2).

Mechanistically, it is unveiled that ultrafine BC causes endothelial senescence and alters the cardiovascular functions at molecular levels (2). Telomerase reverse transcriptase, an enzyme that is required for telomere maintenance, is believed to be critical for proper endothelial cell functions and is inactivated by Src kinase in situations of excessive oxidative stress (2). ufCB increased Src kinase activation and decreased the telomerase activity in lung epithelial and endothelial cells (2). Consequently, ufCB increases senescence of endothelial cells and thus alters cardiovascular functions (2). The data from 642 elderly participated in the Veterans Administration study strongly emphasized the role of soot-mediated cardiovascular diseases. This study demonstrated that BC exposure affected soluble vascular cell adhesion molecule-1 (sVCAM-1), and soluble intercellular adhesion molecule-1 (sICAM-1) both molecule regulate endothelial and cardiovascular system (190). Particularly, diabetics were more sensitive to the BC for both sVCAM-1 and sICAM-1 (190). DEP and CB also altered expressions of cell adhesion molecules and caused oxidative damage in human endothelial cells (191). Proteomic analysis from bronchoalveolar lavage fluid unveiled the action of BC and demonstrated a strong relationship between albumin or alpha2-macroglobulin and vascular endothelial growth factor (151). A difference was also reported in the HR of the soot-exposed mice (70). The other supporting study linked the acute inflammation and cardiovascular functions due to inhalation of diesel and biodiesel exhaust particles (187). Collectively, all studies proved that soot and CB indeed are responsible for the adverse cardiovascular functions. Although these studies show a close association of soot or CB exposure to the cardiovascular symptoms, but the exact mechanism of soot-mediated cardiovascular diseases is not properly understood and needs further investigations.

## The Proposed Mechanisms and Future Perspectives of Soot- and CB-Induced Toxicity

Based on the existing literature discussed in the above sections, the mechanism of soot or CB toxicity can be proposed (Figure 4) (192, 193). Two main kinds of toxicities may exist due to soot or CB exposure: the localized toxicity and the systemic toxicity (194, 195). The localized or direct toxicity includes exposure of soot and its localized (contact mediated) effect by way of oxidative and/or necrotic damage to the lung epithelial cells (161). The second type of toxicity is more systemic in nature and produces toxicity beyond the site of exposure of soot or CB. In fact, systemic toxicity involves immune response that causes damage to the lungs and the other parts of the body (155, 161, 196). However, both types of toxic responses are interlinked and thus soot toxicity can be considered as a combined effect of them.

The localized response of soot is mainly caused by the oxidative stress and dysfunctions in the cellular machinery (Figure 4) (142, 197). One of the mechanisms by which soot or CB exert oxidative stress is the interruption of mitochondrial metabolism (142, 198). This oxidative stress may in turn increase the level of calcium ion release in the cytoplasm and thus affects the cellular signaling (199–202). It is already established that calcium signaling modulates the cell metabolic pathways and alters the cell survival or death pathways (200). Furthermore, it is interesting to know that excessive oxidative stress generated by soot or CB causes extensive DNA damage that leads to the development of cancer (203–205). In fact, oxidative stress produced by the soot interrupts DNA repair machinery and promote cancer like phenomenon (104). Apart from this soot also triggers the cell death probably through the autophagic and apoptotic pathways (132, 206). CB also induces DNA damage and genotoxicity in the *in vitro* cultured cells (99).

**Figure 5:**
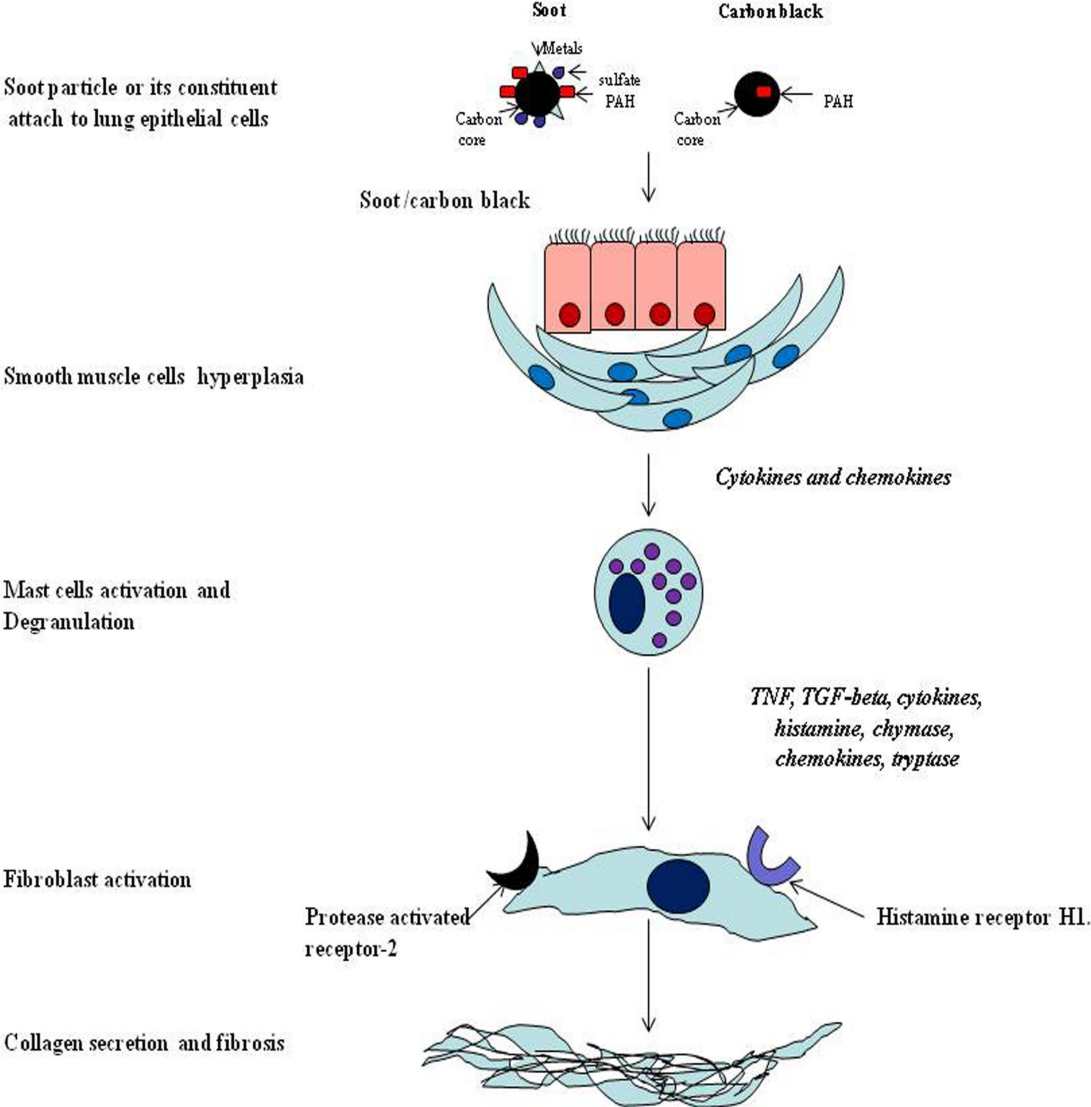
The mechanism of mast cells mediated allergic response due to soot and carbon black. Soot triggers activation of mast cells that in turn release the mediators of inflammation. These mediators subsequently activate fibroblast cells. The fibroblast dysfunctions by these mediators is caused by two receptors (heparin 1 and protease-activated receptor 2) leading to excess production of collagen and other fibrous proteins of the extracellular space.

The systemic response of soot is more complex and affects both local and distal parts of the body by more than one mechanism (133). It is believed that the symptoms of the soot- or CB-induced immune response are similar to allergic diseases (207–209). The CB-induced IgE production in children confirms it as an allergen (210, 211). The subsequent pathologic response *via* IgE pathway is the development of tissue remodeling and fibrosis (209, 212). Importantly, it was noticed that toll-like receptors 2 and 4 are associated with traffic-related air pollutant (soot), which causes development of asthma in children (213). As illustrated in the Figure 4, when soot or CB comes in contact with the lung epithelial cells, various factors are released. These factors attract a huge number of immune cells that damage the lungs tissue in many ways (207, 214). Mainly, eosinophils, natural killer cells [NK and invariant natural killer cells (iNKT)], mast cells, T cells, and innate lymphocyte cells get activated due to the allergic responses (215–217). The blood monocytes and neutrophils may also participate in this initial inflammatory response (217–219). In sharp contrast to the diversity of cell types, mast cells and eosinophils play the critical role in the development of more cumbersome pathogenesis (Figure 5) (220, 221). Eosinophils secrete profibrotic cytokines such as IL-13, IL-4, transforming growth factor (TGF)-, and stem cell factor which in turn induce fibroblast and mast cells proliferation and activation (215, 222, 223). Activated mast cells promote tissue remodeling and fibrosis by secreting tryptase, chymase, and histamine, which work closely with fibroblasts (Figure 5) (215, 224–226). As seen in the figure, monocytes and macrophages become activated and secrete several inflammatory and toxic mediators including MMPs (200, 227–232). Importantly, it is seen that SAA protein is produced in the serum in response to soot (219, 233). It is therefore possible that SAA protein in response to soot exposure may lead to amyloidosis as observed in chronic inflammatory conditions (219, 233). The involvement of Th17 cells are postulated to play a pivotal role in the causation of soot-mediated toxicity (161, 227, 234).

**Figure 6:**
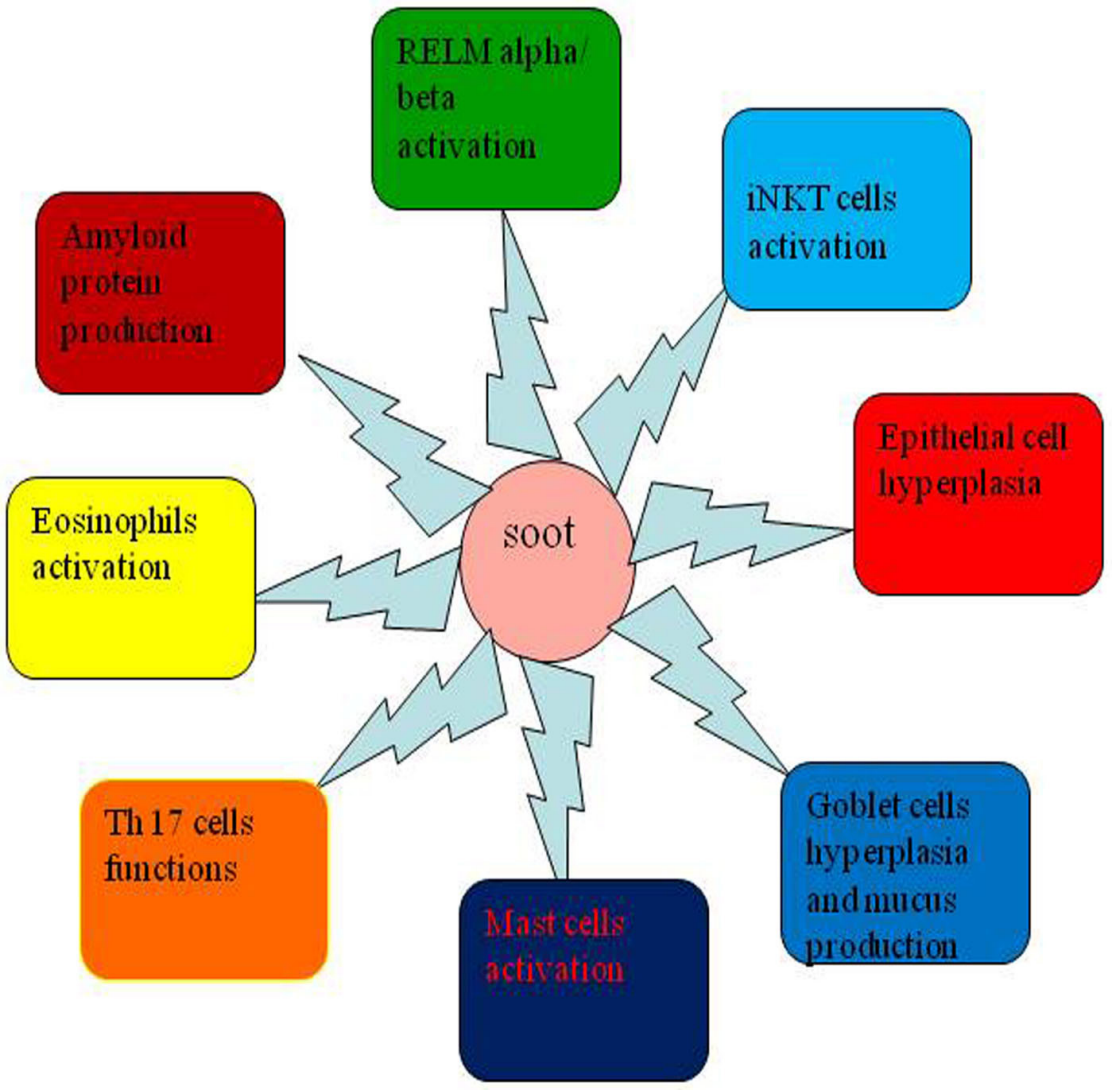
New aspects that need to be explored in the area of soot or carbon black (CB) toxicity. This figure shows new research areas, which need to be explored in soot or CB toxicity. The role of resistin-like molecule-α (RELM-alpha), role of eosinophils and mast cells, activation of invariant natural killer (iNKT) and Th17 cells, and involvement of serum amyloid protein leading to amyloidosis are some of the important areas that still need to be explored in detail.

As of now, a very small part of soot- or CB-induced toxicity is known and a lot more needs to be explored in this area (235). One of the main pathological manifestations of soot or CB is the development of tissue remodeling and fibrosis, the process of which is still largely unknown (161, 236). The fibroblast recruitment to the lung and its role in the soot-induced development of tissue remodeling and fibrosis still remain elusive (212). The role of NK and iNKT cells and eosinophils is not properly known (161, 217, 237, 238). The eosinophils interactions with mast cells are known to modulate the allergic responses in lungs and contribute to the development of tissue remodeling and fibrosis (239, 240). The involvement of chemotactic factors such as eotaxin-1 and eotaxin-2 is also not clear in response to soot or CB exposure (75). A recent break through study described the role of resistin-like molecule alpha and beta (RELM-α/β) in the lung pathology (241). How soot or CB toxicity is associated with the RELM-α/β is not clearly known (Figure 6). The amyloid formation due to inflammatory conditions in lung-related pathologies in response to soot or CB exposure needs exploration in future (242, 243).

## Possible Therapeutic Interventions to Combat Soot- or CB-Associated Disorders

**Figure 7:**
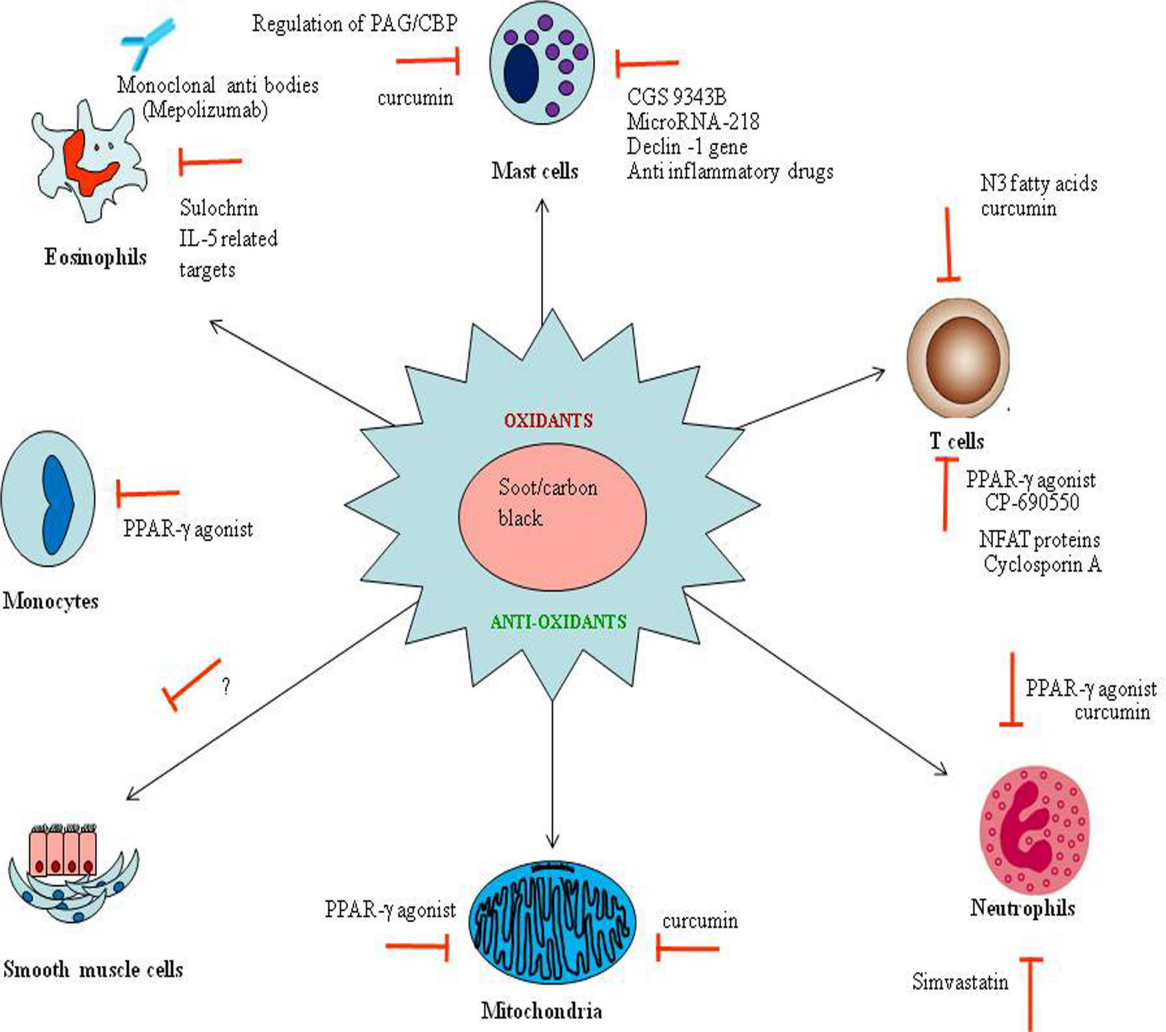
The possible therapeutic strategies to combat soot or carbon black (CB) toxicity. The therapeutic strategies are shown in the figure targeting oxidative stress (in the center) and immune cells (in the periphery) involved in soot or CB toxicity. Red signs show the inhibitors capable of arresting the appropriate response. Anti-eosinophils based antibodies to prevent eosinophils mediated toxic effects are shown. The microRNAs and some noble inhibitors can also be used to downregulate the mast-associated toxicities. N3 fatty acids (polyunsaturated fatty acids) based therapies may be useful to minimize the T cell-associated effects. Importantly, the PPR-γ-based therapeutic interventions become more interesting to target various cell-specific functions.

In recent years, some therapeutic strategies have been suggested to combat the adverse effects of soot or CB (108, 244). As understood from the existing literature and from above discussions, the mechanism of soot toxicity involves immune cells, mediators of inflammation, and various molecules of oxidative stress responsive pathways (245, 246). Therefore, these all may contribute as important targets for the development of novel therapeutics (Figure 7) (247, 248). Here, we discuss some relevant strategies that have been already tested against immune dysfunctions and excessive oxidative stress. Therefore, these can possibly be used to treat soot- or CB-induced toxicities.

The antioxidant therapy can be an important way of treating soot and CB toxicity (249, 250). The existing literature already reported some examples of antioxidant therapy for the pulmonary toxicities (251). Zerumbone, an antioxidant, attenuated Th2 responses induced by ovalbumin and decreased airway inflammation in a mice model of study (252). Similarly, naringin, a flavinoid antioxidant, also attenuated airway inflammation in a mouse model of asthma (253). *Allium cepa* extract and quercetin also showed protective effect in a mice model of asthma (254). *Crocus sativus*, a natural antioxidant, and its main constituents, safranal and crocin, have shown the protective effects against the oxidative stress in the mice model of asthma (255). Resveratrol, a well-known antioxidant, has also shown its protective effects in a mice model of asthma (256–258). The clear example of antioxidant therapy to the soot or CB caused injury came from the effect of artesunate, which significantly decreased the levels of oxidative biomarkers, 3-nitrotyrosine, 8-isoprostane, and 8-OHdG in a mice model of lung injury (259). Similarly, melatonin, a natural antioxidant, was found to reduce airway inflammation in an asthma model (260). Recently, mitochondrial-based antioxidant therapy downregulates TGF-β-mediated deposition of collagen in a murine model of asthma (261). Overall, this can be considered that antioxidant-based therapeutics can be a promising approach against soot- and CB-associated disorders.

It is evidenced that immune cells such as mast cells, eosinophils, T cells, and neutrophils are the major culprit in soot and CB toxicity. Therefore, these may be targeted for the development of noble therapeutic approaches (Figure 7) (215, 226, 262). A monoclonal antibody mepolizumab against the eosinophils activation has been developed and is currently in clinical trials against severe eosinophilic asthma (DREAM) (263). Similar strategies can be used against eosinophils and other mediators of immune response in soot- and CB-mediated toxicity. The mast cells may be the next important target for which a number of therapeutic interventions have been developed (215, 264). Notably, CGS 9343B, a strong inhibitor of calmodulin family, has a potential to inhibit histamine release by mast cells as shown in rats (265, 266). Inhibitors of dectin-1 signaling (R406) downregulated mast cells activation, thus can also be used as a novel therapy to target soot-induced mast cell’s toxicity (267). Similarly, TGaseII/miR-218/-181 can also be used against mast cell activation (Figure 7) (268). Importantly, anti-inflammatory drugs can also interrupt mast cells degranulation and endothelial cells activation (269). Furthermore, a cross-talk between human mast cells and TGF-beta1 signaling has been shown. Therefore, it is postulated to use anti-TGF-beta1 signaling against mast cells associated soot toxicity (270). Recently, a novel, potent dual inhibitor (JNJ-10311795; RWJ-355871) of the leukocyte proteases cathepsin G and chymase has been discovered, which also has an anti-inflammatory activity *in vivo* (271). It has been already reported that cytochalasin B is able to inhibit the cytotoxic response of sensitized lymphoid cells and thus may attenuate mast cells responses (Figure 7) (272). The activation of transmembrane adaptor protein PAG/CBP, which is associated with the both positive and negative regulation cell signaling of mast cells, may also be used as a noble target against mast cells activation (273).

Inhibition of T-cell activation can also be a good idea against soot- or CB-triggered adverse responses (274). It has been reported that PPAR gamma is an important molecule and negatively regulates T cell activation (275). CP-690550 is a Janus kinase inhibitor that downregulate CD4^+^ T-cell-mediated diseases by inhibiting the interferon-gamma pathway (276). Cyclosporin A decreases surface antigen expression on the activated lymphocytes (277). A noble protein “NFAT” has also been reported as a key regulator of T-cell development and functions (278). A unique way of targeting T-cell activation can be the use of N3 fatty acids (omega-3 fatty acids), as they interrupts transcription of human IL-13, which indirectly inhibit the T-helper type 2 effecter immune responses (Figure 7) (279). More recently, the antagonism of noble microRNA-126 has been known to suppress the effecter functions of Th2 cells in the allergic airways disease (280).

Furthermore, inhibition of neutrophils can also be taken into consideration for the development of noble protective strategies. Simvastatin has been shown to affect many adverse functions of neutrophils. In a recent study, it has been shown that simvastatin downregulated secretion of interleukin-8 (IL-8) by neutrophils from the dyslipidemic patients (281). In addition to targeting specialized cells, some other miscellaneous strategies can also be used for the possible management of soot- and CB-associated toxicity. Study conducted in asthmatic rats showed that zinc suppressed inflammation in the airway with exerting effects on the level of eotaxin, IL-8, IL-4, monocyte chemotactic protein-1, and IFN-gamma (282). Curcumin suppresses ovalbumin-induced allergic disease (283). Inhibition of PPR-gamma can be a good strategy for combating the allergic response against soot (284).

## Conclusion and Future Directions

Despite tremendous progress in management of air pollution throughout the world, it continues to harm people’s health and the environment. Nowadays, the problem of air pollution is intensified globally, and soot has been the key pollutant due to its effects on health of humans. The soot and CB toxicity is a broader area of research and should not be only limited to cancer, respiratory, and cardiovascular diseases but also include other disorders. Soot and CB induce cancers at the site of exposure and beyond due to DNA mutations, DNA adducts formation, AhR activation, DNA methylation, and altered oncogenes expressions. The soot- or CB-induced immune response in the lung play critical role in the development of cancer, cardiovascular dysfunctions, and respiratory diseases. The role of eosinophils and other immune cells is critically discussed pointing toward development of noble therapeutics. Based on the existing literature, a consensus mechanism has been proposed depicting the linkages between different cellular and metabolic pathways. As soot and CB can exert chronic inflammatory condition, it may elicit amyloid deposition in different tissues which is largely unknown. The effect of soot on cardiovascular and oxidative stress parameters need to be studied extensively. Altogether, this review provides a better understanding of soot- and CB-induced pathologies and strategies for the possible therapeutics.

## Author Contributions

RN wrote the manuscript. AT was involved in conceptualizing and suggestions and also helped to proof read and organize the final manuscript.

## Conflict of Interest Statement

The authors declare that the research was conducted in the absence of any commercial or financial relationships that could be construed as a potential conflict of interest.

## Abbreviations

PM, particulate matter; DEP, diesel exhaust particles; ROS, reactive oxygen species; TNF-α, tumor necrosis factor-alpha; 8-OHdG, 8-hydroxy-2′-deoxyguanosine; iNKT, invariant natural killer T cells; IL-1, interleukin-1; AD, aerodynamic diameter; ufBC, ultrafine black carbon; PAH, polycyclic aromatic hydrocarbons; CAD, coronary artery disease; CB, carbon black; COPD, coronary occlusion pulmonary disease; Cp, ceruloplasmin; VEGF, vascular endothelial growth factor; ANS, autonomic nerves system; MMP-9, matrix metalloproteases-9; IARC, International Agency for Research on Cancer; UFPs, ultrafine particles; IL-5, interleukin-5; TERT, telomerase reverse transcriptase; sICAM-1, soluble intercellular adhesion molecule-1; Nrf2, nuclear factor erythroid 2; CYP1B1, cytochrome P450 1B1; DE, diesel exhaust; sVCAM-1, soluble vascular cell adhesion molecule-1; RELM, resistin-like molecule alpha and beta; IL-8, interleukin-8; IL-4, interleukin-4; TGF-β, transforming growth factor beta; IL-13, interleukin-13; SCF, stem cell factor; AMPK, adenosine monophosphate-activated protein kinase; SAA, serum amyloid A; MCP-1, monocyte chemotactic protein-1; eNOS, endothelial nitric oxide synthase; NF-kB, nuclear factor kappa B; GM-CSF, granulocyte macrophage colony-stimulating factor; VA, veteran administration; PUFAs, polyunsaturated fatty acids; NK, natural killer cells.
